# Forgotten Features of Head Zones and Their Relation to Diagnostically Relevant Acupuncture Points

**DOI:** 10.1093/ecam/nen088

**Published:** 2010-10-19

**Authors:** Florian Beissner, Christian Henke, Paul U. Unschuld

**Affiliations:** ^1^Brain Imaging Center, Goethe-University, Schleusenweg 2-16, 60528 Frankfurt, Frankfurt, Germany; ^2^Institute of Neuroradiology, Goethe-University, Schleusenweg 2-16, 60528 Frankfurt, Frankfurt, Germany; ^3^Department of Neurology, Goethe-University, Schleusenweg 2-16, 60528 Frankfurt, Frankfurt, Germany; ^4^Horst Goertz Institute for Theory, History and Ethics of Chinese Life Sciences, Charité University Medicine, Berlin, Germany

## Abstract

In the 1890s Sir Henry Head discovered certain areas of the skin that develop tenderness (allodynia) in the course of visceral disease. These areas were later termed “Head zones”. In addition, he also emphasized the existence of specific points within these zones, that he called “maximum points”, a finding that seems to be almost forgotten today. We hypothesized that two important groups of acupuncture points, the diagnostically relevant Mu and Shu points, spatially and functionally coincide with these maximum points to a large extent. A comparison of Head's papers with the Huang Di Neijing (Yellow Thearch's Inner Classic) and the Zhen Jiu Jia Yi Jing (Systematic Classic of Acupuncture and Moxibustion), two of the oldest still extant Chinese sources on acupuncture, revealed astonishing parallels between the two concepts regarding both point locations and functional aspects. These findings suggest that the Chinese discovery of viscerocutaneous reflexes preceded the discovery in the West by more than 2000 years. Furthermore, the fact that Chinese medicine uses Mu and Shu points not only diagnostically but also therapeutically may give us new insights into the underlying mechanisms of acupuncture.

## 1. Introduction

### 1.1. Anatomical Correlates of Acupuncture-Related Structures

The search for anatomical correlates of acupuncture-related structures (acupoints, conduits, etc.) has been an ongoing effort since the very first days of acupuncture research. The anatomical structures most often investigated in this context are probably those of the nervous system. This popularity is easily understandable because the nervous system offers mechanisms for both afferent and efferent transmission of signals. A stimulus delivered by an acupuncture needle anywhere to the body can irritate local nerve fibers and elicit afferent signals that run to some central relay station (spinal cord, brain). Here the signals can cause the excitation of an efferent nerve that can travel to virtually any part of the body, where it can produce a large variety of effects. Virtually all those functions in the human body influenceable by acupuncture are neuronally regulated, for example, pain modulation [1, 2], immune system control [3, 4], inner organ functions [5, 6], endocrine functions [7], and many more. Thus disregarding all the intricate details of neuronal pathways and connections the nervous system theoretically offers everything needed to explain the effects usually seen in acupuncture treatments.

### 1.2. Head Zones

An important neuronal concept is the so-called Head zones, discovered more than 100 years ago by Sir Henry Head (1861–1940). In a seminal series of papers he published data collected on hundreds of clinical cases [8–11]. In his studies, Head pursued a 2-fold approach comparing areas of cutaneous tenderness (i.e., dynamic or thermal allodynia) in viscerally diseased patients with patterns observed in rashes of herpes zoster (shingles). Today Head zones are thought to coincide to a large extent with dermatomes [12], that is, areas of skin innervated by one and the same spinal nerve. The most often cited theory for the mechanism of Head zones is that of viscerocutaneous reflexes: Viscero- and somatoafferent (nociceptive) neurons converge on the level of the spinal cord. This convergence is thought to take place near the lateral column, although the exact location in terms of Rexed laminae as well as the mechanism leading to the false reference of visceral to cutaneous pain signals are still unknown. It is interesting to note that such a common diagnostic tool as the Head zones that every medical student learns during his studies, is still so poorly understood more than 100 years after its discovery [13]. Also on the macroscopic scale there remain a lot of unanswered questions, for example, the exact locations of the zones and their degree of overlap [14] as well as their mutual functional interactions [15].

### 1.3. Mu and Shu Points

The Chinese concepts of frontal Mu points and Shu points of the back date back to the very beginning of acupuncture. Both can be found in the Huang Di Neijing (Yellow Thearch's Inner Classic) text corpus, the oldest still extant source of Chinese medicine containing information on acupuncture. The Huang Di Neijing is based on numerous small writings dating from the second and first centuries BCE and possibly the first and second centuries CE. These brief texts appear to have served as a pool from which various compilers or compiler teams drew their materials in perhaps the second or third centuries CE when they prepared what became later known as Huang Di Neijing Suwen (Basic Questions), Huang Di Neijing Lingshu (Spiritual Pivot), Nanjing (Classic of Difficult Issues) and Shanghan Lun (On Harm Caused by Cold) [16]. The terms “Mu” and “Shu” convey the meanings of “to levy” and “to transport”, respectively. They are one example of several terminological pairs reflecting assumed yin/yang dualism in the organism, one of the conceptual foundations of Chinese medicine. In modern TCM textbooks, Mu points are often called “alarm points”, a mistranslation that hints at the diagnostic relevance of these points. For the sake of completeness, it should be noted that there are also other points in Chinese medicine besides Mu and Shu points that have a close relation to inner organs. Two examples are the so called Xi (“cleft”) and Xiahe (“lower see”) points.

In the following text, we will review the works of Head alongside with some classics of Chinese medicine in order to check for similarities between the two areas. An extensive review of existent (English) literature revealed that over the past three decades many authors have proposed connections between Head zones and acupoints (see, e.g., [17–19]). Even more works have focussed on parallels with dermatomes and segmental innervation, respectively. Although this connection seems to be well established, none of the works explicitly focussed on the so-called “maximum points” of the Head zones. We hypothesized that the diagnostically relevant Mu and Shu points, coincide to a large extent with these maximum points.

## 2. Methods

### 2.1. Maximum Points of Head Zones—A Forgotten Feature?

**Figure 1:**
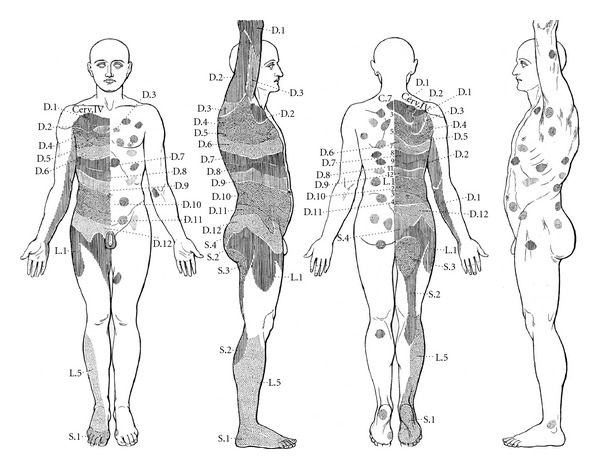
Original drawing from Head's first paper showing Head zones together with maximum points (from [8, pages 131-132], by permission of Oxford University Press).

A feature of Head's work that seems to have been totally forgotten are what he called “maximum points”. It seems that Head himself found this discovery much more important than the zones in general (see statement in Table1(a)). Furthermore, he reports the maximum points to coincide with those areas, where the first blisters appear in a rash of herpes zoster, and where they start to spread from. Consequently, he depicts these maximum points alongside with the zones in his first paper: Figure 1 shows a slightly modified version of Head's original drawings showing the zones on one and the maximum points on the other side of the body. Surprisingly, this part of his work is almost never cited. A possible explanation for this may be that in contrast to the zones, which can be partly explained by segmental innervation, no such anatomical explanation exists for Head's maximum points so far.

### 2.2. Localization of Mu and Shu Points by Ancient Literature Sources

#### 2.2.1. Shu Points

**Figure 2:**
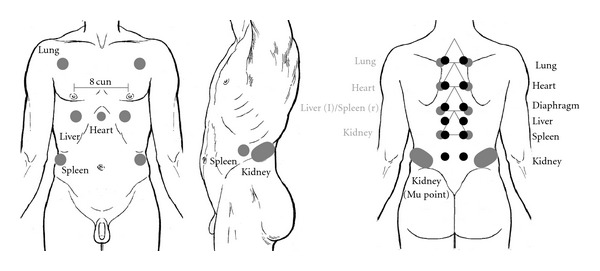
Locations of Mu and Shu points superimposed on a torso from Head's paper [8, pages. 131-132], by permission of Oxford University Press. The Mu point locations are according to the Zhen Jiu Jia Yi Jing. For the Shu points gray indicates locations according to the Suwen, black according to the Lingshu.

Shu points are the only points mentioned in the Huang Di Neijing Suwen that are given with a clear anatomical description (see Table 2(a)). Because the author uses a proportional measure here, namely the distance between the nipples, we can easily localize the Shu points on a drawing similar to Head's original one (see Figure 2). Not unusual for this early state of Chinese medical history the Lingshu gives a different description of the Shu points (Table 2(b)). Here the author uses vertebrae as anatomical landmarks as well as a unit called cun. This is the main proportional measurement unit of Chinese medicine. One cun equals the width of the patient's thumb making the distance between the nipples ~8 cun (see Figure 2). There is a marked difference for the points of the liver, spleen and kidneys. The different locations are shown in Figure 2. In later times, more and more Shu points were added to this list. However, we will stick to those for the five so called “Zang” literally meaning “depots” (i.e., places of Yin nature where important items are stored for long: lung, heart, liver, spleen, and kidneys) [16]. This is for the sake of simplicity, because we are interested in the question, how ancient Chinese doctors found out about these points in the first place knowing that there was a general tendency in later times to make all findings fit into the theory of systematic correspondences, for example, the five phases doctrine [20], possibly obscuring the view on the original discovery.

#### 2.2.2. Mu Points

Although the Huang Di Neijing Suwen mentions the Mu points of the stomach (chapter 28) and the gallbladder (chapter 47) the first source compiling Mu points as a group is the Mai Jing [(Movements in the) Vessels Classic] by Wang Shu-He from the third century CE. Therein the Mu points of liver, gallbladder, heart, small intestine, spleen, stomach, lung, large intestine, kidneys and bladder are presented. Another work of that time, the Zhen Jiu Jia Yi Jing (Systematic Classic of Acupuncture and Moxibustion) by Huang-Fu Mi describes for the first time the anatomical locations of these points.

Here again we will only include those Mu points in our comparison that correspond to the five depots. They are depicted in Figure 2. Locating the Mu points by the descriptions of Huang-Fu was quite easy for lung, heart, liver and spleen due to the anatomical landmarks given (for detailed descriptions see the Supplementary Material). However, locating the kidney Shu point turned out to be difficult. For a description of the localization by Huang-Fu see Table 2(c). We used larger points in Figure 2 to account for the uncertainty in location.

## 3. Results

### 3.1. Comparing Mu and Shu Points with Head's Maximum Points

In the following text, we will compare Head's results with the data recorded in ancient Chinese medical texts. Our hypothesis is that Mu as well as Shu points coincide to a large extent with Head's maximum points. To prove this, we will first look for similarities in the definitions of these entities and then review some clinical cases from Head's papers. The functional properties of Shu points and the indication for their therapeutic use are well described in the Lingshu (Table 2(d)). Most interestingly, Head reports a very similar finding in a case of incurable diarrhoea, where the patient developed cutaneous tenderness on the abdomen and the back.

Since Head in the 1890s did not use any diagnostic technique other than palpation and interview from this point of view it is well imaginable that Chinese doctors were able to find the same results. The reader may judge for himself when comparing Figure 1 with Figure 2. Because of the large number of maximum points and their more or less homogeneous distribution over the body, it may be too tempting to see correspondences where there are none. Therefore, we chose to take single cases reported by Head and to compare their maximum points to the expected painful Mu and Shu points in this disease.

### 3.2. Clinical Cases from Head's Paper

**Figure 3:**
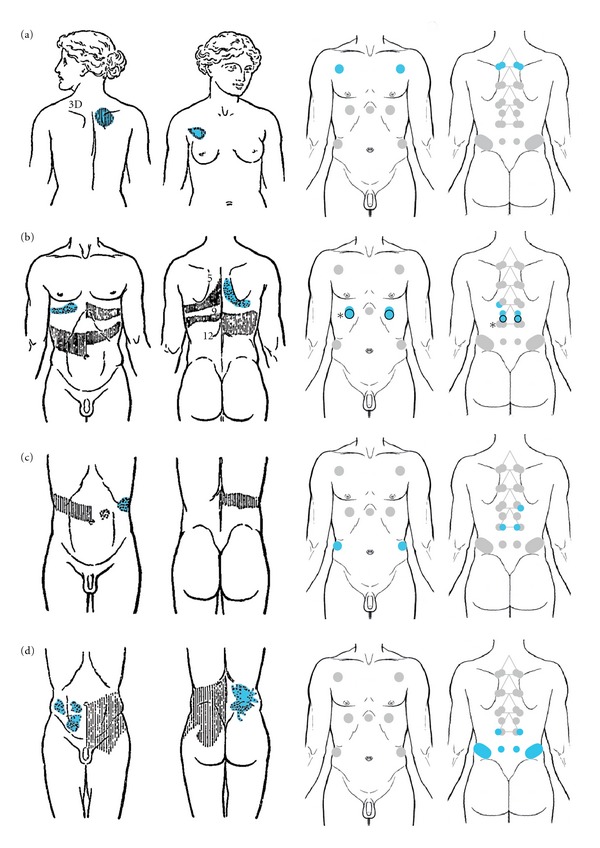
Comparison of four clinical cases from Head's first paper showing their areas of cutaneous tenderness (left) with the corresponding Mu and Shu points from the viewpoint of Chinese medicine (right). The maximum points, Mu and Shu points are marked in blue. The patients depicted here all have a diseased organ: (a) Lung, (b) Liver, (c) Stomach, (d) Kidney/Ureter and were all taken from [8] (with permission of Oxford University Press).

The cases we chose for the evaluation can be frequently seen in hospitals these days. We can expect they were also regularly observed 2000 years ago in China. For the selection of cases, we restricted ourselves to Head's first paper [8] because the others cover only special aspects of the whole topic. This paper covers 41 cases given with description and picture. Of these 41 cases, 16 show rashes of herpes zoster while four show disturbances of sensation due to neurological problems. Furthermore, there are four cases of systemic infectious diseases (influenza, typhoid fever, small pox). Twelve cases exhibit diseases of organs not covered in our restriction to depot organs and one shows a multi-organ disease. The remaining four cases are shown on the left side of Figure 3. Since Head always reports areas of allodynia together with the maximum points, the latter are accentuated in blue. The right side shows the Mu and Shu points associated with the organ(s) affected by the respective disease. Since medicine has advanced in the last 110 years, one of the authors (C.H.) has re-evaluated Head's diagnoses together with another physician based on the symptoms reported in the paper. Whenever necessary, the diagnosis has been altered to match today's knowledge and terminology.

Patient (a) is case no. 24 (p. 61). The woman suffers from an acute bronchitis. This is one of the most obvious examples because there are no areas of allodynia besides the maximum points themselves. The Mu and Shu points marked in blue on the right hand side correspond nicely to this pattern.

Patient (b), case no. 31 (p. 74) suffers from gall bladder stones manifesting as gallstone colics. He has also been diagnosed with a Klatskin tumor post-mortem: “*Bile ducts surrounded by dense firm tissue. At portal fissure large mass of new growth. Growth limited very closely to Glissons capsule.”* The Mu and Shu points we would expect are those of the liver. Again, we see nice correspondence. One may argue that this is more a gallbladder than a liver problem. Disregarding our restriction to those points of the depots, we have added them to our display. They are marked with asterisks and lie in very close vicinity to those of the liver according to the descriptions of Huang-Fu (see Supplementary Material).

For patient (c), case no. 27 (p. 67) Head reports: “*Pain in the abdomen for the last twelve hours. Intense—keeps him doubled up. […] Bowels open. No constipation or diarrhoea. Has vomited several times. Pain and tenderness gone next day.”* Retrospectively we can only follow Head and describe this sickness as a gastritis. The corresponding Mu and Shu points can be clearly identified as those of the spleen. This would lead to a clearly wrong diagnosis from the viewpoint of Western medicine. However, in the classics of Chinese medicine we can find that the spleen is thought to control digestion (see Table 2(e)). According to this statement of the Suwen, the spleen receives the ingested substances from the stomach to extract the nutrients. In fact if one asks a practitioner of Chinese medicine today, one would still get the answer that there is an important functional involvement of the spleen in digestion.

Patient (d) shows case no. 32 (p. 77) suffering from nephrolithiasis with a single ureter stone. Head comments on the rather large area of the maximum point(s): “*It is interesting to note that this case showed a rather wider distribution downwards [11th and 12th dorsal] than is usual in cases of renal calculus [*…*]”* but clearly points out a maximum point: “*The tenderness is especially marked over the tip of the twelfth rib [*…*]”* Again there is at least a large overlap between the maximum points and the locations of the Shu as well as the Mu points of the kidneys.

We can state that for all cases from Head's paper [8] showing diseases of “depot” organs the maximum points correspond nicely with the expected Mu and Shu points.

However, as the reader may have noticed, we have not included a case of heart disease in our comparison. This is because Head's paper does not include such cases, but only two cases of aortic diseases, which is no heart disease but a vascular problem. There may be several reasons for this lack in Head's paper. Firstly, the cutaneous tenderness and referred pain in acute heart attacks (like angina pectoris) is often very widespread making it difficult to define maximum points. Secondly, one of his colleagues, James MacKenzie (1853–1925), was working on the same phenomenon mainly in cases of heart diseases [21, 22], and it may well be that Head did not want to interfere too much with him. In heart attacks, one mainly finds pain in the chest in addition to the well known radiating pain in the arm, which by the way follows exactly the course of the heart conduit of Chinese medicine as has been stated before numerous times. However, also the Mu and Shu points of the heart can be found in cases of heart disease as a look into MacKenzie's work [21] reveals (p. 17, Figure 1 and p. 19, Figure 4).

## 4. Discussion

When investigating the parallels of Western and Chinese ideas, we were astonished by the high degree of correspondence between these two systems. It is more than just historically interesting that the Chinese discovery of viscerocutaneous reflexes and their underlying systematics probably preceded the discovery in the West by more than 2000 years. Additionally one may ask, whether there is something more we can learn for today's medicine.

The most important difference between Western and Chinese concepts is that in the West Head zones are purely used as a diagnostic tool. In all cases, the diagnosis will be checked by means of more advanced techniques. In Chinese medicine, however, Mu and Shu points are both, diagnostically and therapeutically relevant. When a point is aching or when pressure on the point relieves an existing pain, this point is considered for treatment with acupuncture, moxibustion or related techniques. It is this simple idea to take a reversed action—from the skin to the viscera—for granted, which makes the Chinese concept so intriguing [18]. All techniques used to treat these points (e.g., acupuncture) involve some kind of sensory or even pain stimulation. And because the exact mechanism behind viscerocutaneous reflexes is far from being fully understood, one could well imagine such an action. Again Head seems to have had the same idea already, when he described the use of maximum points for treatment (Table 1(c)). The mustard oil mentioned here is known to cause pain by activating TRPA1 receptors [23] thus Head obviously successfully applied a kind of sensory stimulation therapy using irritant substances on the points he just discovered. However, this idea, to use maximum points therapeutically, has been lost in conventional medicine today, while similar concepts are in use in a variety of alternative medical treatments.

We think that an effort should be made to elucidate the exact mechanisms behind Head zones. Their diagnostic as well as their therapeutic potential should be re-evaluated clinically. A next question could be for example, whether acupoints corresponding to maximum points also have stronger therapeutic effects than others. Finally Mu and Shu points could be an important starting point in the understanding of the underlying mechanisms of acupuncture.

## Supplementary Material

Descriptions of Mu point locations from the Zhen Jiu Jia Yi Jing (Systematic Classic of Acupuncture and Moxibustion) by Huang-Fu Mi, and description of Shu point locations from the Mai Jing ([Movements in the] Vessel Classic) by Wang Shu-He.

## Figures and Tables

**Table 1:** Head's statements on maximum points.

(a) “*Every such area of cutaneous tenderness has one or more maximum points, the position of which is exceedingly important, for it is to the situation of these maxima that the patient refers his pain*”. Head [8, page 6]	
(b) “*There is great cutaneous tenderness […] Yet firm deep pressure relieves, rather than aggravates, his pain*”. Head [8, page 71]	
(c) “*Thus, mustard leaves applied to the maximum spots of the affected areas of the chest or back, […] will remove the nausea and vomiting in this mild and purely reflex type of gastric disturbance*”. Head [8, page 261]	

**Table 2:** Statements from two Chinese classics (Huang Di Neijing and Zhen Jiu Jia Yi Jing) on Mu and Shu points.

(All translations by P. U. Unschuld)	
(a) “*If one wishes to know [the location of] the transporters on the back, one first measures the distance between the two breast nipples [with a stalk of grass]. One breaks [this stalk] in the middle. Again one takes another [stalk of] grass, measuring [the same distance], and removes one half of it. […] Then one lifts [the resulting triangle] to measure this [person' s] back. One lets one angle be situated upwards, on the same level as the Great Hammer [hole] on the spine. […] Exactly at the location of the lower angles are the transporters of the lung. One measurement further down, are the transporters of the heart. One measurement further down, at the left angle is the transporter of the liver; at the right angle is the transporter of the spleen. One measurement further down are the transporters of the kidneys. These [locations] are called “the transporters of the five depots*”. Huang Di Neijing, Suwen, chapter 24. Comment: Great hammer in this context refers to the acupoint Dazhui, situated in a depression directly below the spinous process of the 7th cervical vertebra.	
(b) “*The great transport [location for qi] in the chest is at the tip of the shuttle bone. The transport [location for qi] of the lung is located to the side of the third vertebra. The transport [location for qi] of the heart is located to the side of the fifth vertebra. The transport [location for qi] of the diaphragm is located to the side of the seventh vertebra. The transport [location for qi] of the liver is located to the side of the ninth vertebra. The transport [location for qi] of the spleen is located to the side of the eleventh vertebra. The transport [location for qi] of the kidneys is located to the side of the fourteenth vertebra. All are located on both sides of the spine. Their distance from each other is 3 cun*”. Huang Di Neijing, Lingshu, chapter 51	
(c) “*Capital Gate is the gathering [hole] of the kidney [*…*]. It is located below the hip bone in the lumbar region lateral to the spine, one cun and eight fen below the region of the free ribs*”. Zhen Jiu Jia Yi Jing, book 3, chapter 23	
(d) “*If one wishes to successfully employ them, one must press these locations. There will be a response inside, and the pain [felt by the patient] is ended*”. Huang Di Neijing, Lingshu, chapter 51	
(e) “*Now, the five flavours enter the mouth, and they are stored in the stomach. The spleen moves the essence qi on behalf of the [stomach]*”. Huang Di Neijing Suwen, chapter 47	
