# Supplementary material for: Forgotten Features of Head Zones and Their Relation to Diagnostically Relevant Acupuncture Points
Source: Evid Based Complement Alternat Med. 2010 Oct 19;2011:240653. doi: 10.1093/ecam/nen088 (PMC3135114; doi:10.1093/ecam/nen088)
Supplement: Supplementary file 1 — Descriptions of Mu point locations from the Zhen Jiu Jia Yi Jing (Systematic Classic of Acupuncture and Moxibustion) by Huang-Fu Mi, and description of Shu point locations from the Mai Jing ([Movements in the] Vessel Classic) by Wang Shu-He. [file 240653.f1.pdf]

Descriptions of Mu point locations from the Zhen Jiu Jia Yi Jing (Systematic Classic of Acupuncture and Moxibustion) by Huang-Fu Mi:

“Camphorwood Gate is the levy [hole] of the spleen [...]. It is located outside the Great Horizontal, directly [level] with the umbilicus at the end of a free rib [...].”
(The point Great Horizontal mentioned as a landmark here lies on one level with the umbilicus on the mamillary line.)

“Central Treasury is the levy [hole] of the lung [...]. It is located one cun below Cloud Gate in a depression in the breast above the third rip where the pulsation of a vessel is palpable.”
(The point Cloud Gate is located in a depression directly below the clavicula.)

“Great Tower Gate is the levy [hole] of the heart and is located one cun below Turtledove Tail.”
(The point Turtledove Tail is located 5 fen below the xiphoid process.)

„Cycle Gate is the levy [hole] of the liver. It is located 1.5 cun from both the tip of the angle of the second rip and Not Contained directly inferior to the breast [...]“
(The point Not Contained is located 3 cun 5 fen from Great Gateway, which lies on the middle line 6 cun above the umbilicus.)

”Sun and Moon is the levy [hole] of the gallbladder. It is located five fen below Cycle Gate [...].”

Description of Shu point locations from the Mai Jing ([Movements in the] Vessel Classic) by Wang Shu-He

> *“The transport [hole] of the gallbladder is located at the tenth vertebra in the back [...]”*
>
> *“The transport [hole] of the large intestine is located at the sixteenth vertebra in the back [...]”*
